# Performance Analysis of the SIFT Operator for Automatic Feature Extraction and Matching in Photogrammetric Applications

**DOI:** 10.3390/s90503745

**Published:** 2009-05-18

**Authors:** Andrea Lingua, Davide Marenchino, Francesco Nex

**Affiliations:** Politecnico di Torino, DITAG, C.so Duca degli Abruzzi, 24 – 10129, Torino, Italy; E-Mails: andrea.lingua@polito.it (A.L.); francesco.nex@polito.it (F.N.)

**Keywords:** feature extraction, feature matching, image orientation, SIFT operator, location accuracy

## Abstract

In the photogrammetry field, interest in region detectors, which are widely used in Computer Vision, is quickly increasing due to the availability of new techniques. Images acquired by Mobile Mapping Technology, Oblique Photogrammetric Cameras or Unmanned Aerial Vehicles do not observe normal acquisition conditions. Feature extraction and matching techniques, which are traditionally used in photogrammetry, are usually inefficient for these applications as they are unable to provide reliable results under extreme geometrical conditions (convergent taking geometry, strong affine transformations, etc.) and for bad-textured images. A performance analysis of the SIFT technique in aerial and close-range photogrammetric applications is presented in this paper. The goal is to establish the suitability of the SIFT technique for automatic tie point extraction and approximate DSM (Digital Surface Model) generation. First, the performances of the SIFT operator have been compared with those provided by feature extraction and matching techniques used in photogrammetry. All these techniques have been implemented by the authors and validated on aerial and terrestrial images. Moreover, an auto-adaptive version of the SIFT operator has been developed, in order to improve the performances of the SIFT detector in relation to the texture of the images. The Auto-Adaptive SIFT operator (A^2^ SIFT) has been validated on several aerial images, with particular attention to large scale aerial images acquired using mini-UAV systems.

## 1. Introduction and State of the Art

Feature extraction is one of the main topics in Photogrammetry and Computer Vision (CV). This process consists of the extraction of features of interest from two or more images of the same object and of the matching of these features in adjacent images.

In aerial and close-range photogrammetry, image features are necessary for automatic collimation procedures such as image orientation, DSM generation, 3D reconstruction, and motion tracking. In CV, features are used in various applications including: model based recognition, texture recognition, robot localization [1], 3D scene modelling [2], building panoramas [3], symmetry detection and object categorization. In the last 25 years, many photogrammetric and CV applications dealing with feature extraction have been developed. Photogrammetric research has led to feature extraction algorithms, called interest operators or point detectors, while many region detector techniques have been developed in CV.

Interest operators extract salient image features, which are distinctive in their neighbourhood and are reproduced in corresponding images in a similar way [4]; at the same time, interest operators supply one or more characteristics, which can be used in the image matching. Region detector operators, instead, search for a set of pixels which are invariant to a class of transformations (radiometric and geometric distortions). The term ‘region’ differs from classical segmentation since the region boundaries do not have to correspond to changes in image appearance such as colour or texture [5].

These operators have been developed for when the normal stereo image acquisition condition is not required. Region operators detect features that do not vary with different geometrical transformations (scale, affine transformation, etc.). A descriptor, which describes the extracted feature using a 2D vector that contains gradient pixel intensity information, is associated to each region. This information may be used to classify the extracted regions or to perform the matching process.

Although region detector/descriptors are computationally slower than those of interest points, the experimental results show that these detectors have a wider application range. Interest in these detectors in the photogrammetric field is quickly increasing due to the introduction of new image acquisition techniques, which do not comply with the normal stereoscopic case. Images acquired through Mobile Mapping Technology [6] are usually extracted from video-sequences with low-resolution quality. Consequently, the orientation process is hampered by illumination problems, the limited dynamic range of the video-cameras, sensor noise, narrow baselines and projective distortions. Oblique photogrammetric images, which are commonly used for the generation of 3D city modelling [7], offer images that are affected by high projective distortions which must be carefully processed. Finally, image sequences acquired using low-cost UAV platforms [8] do not assure the normal taking geometry.

Interest point extractors and matchers, which are traditionally used in photogrammetry (Forstner operator [9], Harris operator [10], Cross-Correlation etc.), are usually inefficient for these applications as they are unable to give reliable results under difficult geometrical and radiometrical conditions (convergent taking geometry, strong affine transformations, lack of texture etc.)

The SIFT operator is one of the most frequently used in the region detector field. It was first conceived by Lowe [11] and it is currently employed in different application fields. In CV SIFT has been used for object retrieval [12], 3D matching [13], 3D scene reconstruction [14], robot localization and mapping [15], action recognition [16], panorama stitching [17] and motion tracking [18-19]. SIFT has also been applied in Photogrammetry, in close-range applications, for 3D modelling of small objects [20] and for spatio-temporal feature tracking analysis [21]. Moreover, SIFT has also been applied in remote sensing [22-23], in the registration of LIDAR intensity data and aerial images [24], in the co-registration of synthetic aperture radar interferometry data [25] and in real-time mapping applications from UAV [26]. Several methods similar to the SIFT operator method have been developed in order to overcome its high computational cost; however, faster implementations (PCA [27], SURF [28], etc.) reduce the point location accuracy.

Although many papers and much research about feature detectors have been carried out within the CV community, detailed studies concerning the accuracy of the SIFT operator have never been performed in the Photogrammetry field. Some articles which compare feature detectors can already be found in literature: Mikolajczyk [5-29] has analysed the performances of affine-invariant and scale invariant region detectors and Schmid [30] has evaluated the performances of interest point detectors. These papers evaluate the feature extractors in terms of the number of extracted points and repeatability and show that the SIFT detector supply more stable results than the other ones. However, the determination of the localization accuracy has only been performed on terrestrial images.

Accuracy is the most important criterion for the evaluation of a good photogrammetric process. For this reason, the main goal of researchers in photogrammetry is to assess the accuracy that feature points and region operators can reach in the automatic feature extraction and matching phases of the photogrammetric process. Remondino [31] has carried out tests on six regions and interest point detectors. He has compared the results obtained from a quantitative analysis that was based on the relative orientation between image pairs. The test results, highlighted optimal performances of the region detectors (in particular SIFT) as far as the number of points extracted is concerned, even though the accuracy was not as high as that of the interest operator ones. Furthermore, the author showed that the accuracy of SIFT can be improved using the Least Square Matching (LSM) algorithm [32]. However, only a SIFT demo-version was dealt with in this paper and only terrestrial images were considered.

The performance analyses performed in the previous researches on the SIFT technique have dealt with the geometric and the illumination conditions of the image acquisition, but they did not consider the dynamic range of the image or the texture distribution. In [18] the importance of contrast thresholds of the SIFT in relation to the number of extracted points has been underlined. This aspect influences the performances of the SIFT detector, especially in aerial applications over non-urban areas, such as grasslands, ploughed fields or wooded zones. In these cases, the local dynamic range of the image is quite low and the image can be defined “bad textured”. Therefore, some threshold parameters proposed by Lowe [10] for the removal of low-contrast regions have to be corrected.

In the first part of the paper, an assessment has been made of SIFT performances on terrestrial and aerial images. The goal is to evaluate of the potential of SIFT with respect to some photogrammetric process phases, such as automatic tie point extraction and approximate DSM generation. Tie-points are useful in the image orientation and they provide an approximate DSM that is essential for several image matching algorithms [33]. These algorithms usually extract an approximate DSM through a pyramid approach [34]; although, this approach is useless if a high number of points has already been extracted by SIFT.

For this reason, a complete and reliable comparison between the SIFT operator and traditional photogrammetric feature extraction and matching techniques has been carried out. SIFT, Forstner operator, Cross-Correlation and Least Square Matching techniques have been implemented and validated on different images. Synthetic, terrestrial and aerial images, acquired under non-normal conditions (rotation, scale change, convergent taking geometry), have been considered. The analysis was performed using the local accuracy criterion and evaluating the number of homologous points extracted.

Then, the performance of the SIFT detector has also been assessed in relation to the texture of the images. A modified version of the SIFT detector has been developed and implemented for this purpose. The Auto-Adaptive SIFT (A^2^ SIFT) allows the contrast threshold parameters of the SIFT detector to be defined, in relation to the local texture around each feature. Some experimental tests on aerial images acquired using a mini-UAV system show that A^2^ SIFT allows the feature extraction and matching to be increased, especially on areas with a high rate of repetitive-patterns or bad textures.

In the following sections the algorithms, the testing methodology and the achieved results will be presented in more detail.

## 2. SIFT Operator

SIFT (Scale Invariant Feature Transform) is a region detector/descriptor [5-35] which extracts image features that are invariant to image scaling and rotation and partially invariant to changes in illumination and the 3D camera viewpoint (affine transformation). A vector (dimension 128), called descriptor, is associated to each feature. This vector allows the feature matching between image pairs that satisfy the previously mentioned geometrical transformation to be carried out.

Feature detection is performed using a staged filtering approach, according to the theoretical and experimental results of Koenderink [36] and Lindeberg [37]. The assumption is that the only possible scale invariant image space is given by a Gaussian function. The scale-space is defined as a function *L*(*x, y, σ*) which is produced from the convolution of a Gaussian kernel *G*(*x, y, σ*) (Equation 1) with the input image *I*(*x, y*):

(1) L(x,y,σ)=G(x,y,σ)∗I(x,y)

Lowe proposed a new method for the detection of scale-invariant keypoints. As far as the Lindeberg algorithm is concerned, SIFT extracts keypoints as the maximum response of the Difference-of-Gaussian (DoG) function, which can be computed from the difference of two nearby scales *s* separated by a constant multiplicative factor *k*:

(2) $$\begin{array}{l} D\left(x,y,\sigma \right)=\left(G\left(x,y,k\sigma \right)-G\left(x,y,\sigma \right)\right)*I\left(x,y\right)\\ \;=L\left(x,y,k\sigma \right)-L\left(x,y,\sigma \right) \end{array}$$

The main advantage of Lowe's detector is the computational cost, which is reduced compared to the Lindeberg method. This is due to the replacement of the scale-normalized Laplacian of Gauss with a difference of Gaussian functions, which is computed with a simple image subtraction.

Feature points are detected in the DoG scale-space as the local maxima and minima of *D(x, y, σ)*. At a given image scale, each image point is compared to the eight adjacent pixels and the nine neighbours in the scale above and below. Local maxima or minima are classified as keypoints. In order to select stable keypoints, the local extrema values of *D(x, y, σ)* must be higher than a threshold *(Th_key)*.

The local detection is refined using a sub-pixel localization approach, developed by Brown [38]. Local maxima can be detected by fitting the 3D quadratic function *D(x, y, σ)* to the local sampled point by means of a Taylor expansion [35].

The stability of the local detection is improved by the removal of keypoints with low contrast. The scale-space *D(x, y, σ)* computed at the sample point is, in particular, useful for removing low-contrast extrema. The threshold value proposed by Lowe is. D|(*x̂*| =0.03 This value is assessed according to experimental results on images with a high local dynamic range (“well textured” images).

The DoG scale-space supplies keypoints that are invariant to scale transformation, but sensitive to rotation. In order to avoid these problems during feature matching operations, the SIFT detector assigns a canonical orientation at each keypoint, based on the local radiometric properties of the neighbouring pixels.

The image matching between the keypoints extracted in a stereopair is carried out using the SIFT descriptor. This matching has the goal of “describing” a local image region around keypoints in a manner that is invariant to the scale and rotation. The SIFT descriptor belongs to the class of Distribution Based Descriptors (DBD). These techniques use histograms to represent different characteristics of the appearance and shape. A simple descriptor is the distribution of the pixel intensities represented by a histogram. More expressive and complex representations are described in [39] and [40].

The great advantage of this operator is its robustness against small shifts in the local geometry, such as those that arise from affine or homographic projection.

**Figure 1.**
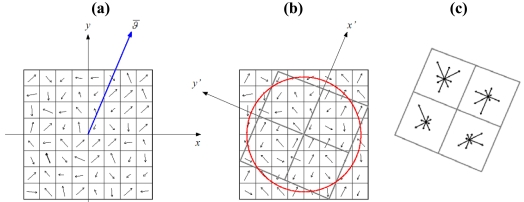
(a) The SIFT descriptor. Computation of the magnitude and orientation. (b) Orientation corrections and spatial coordinate transformation. (c) SIFT descriptor establishment. In the original implementation, there are 16 spatial bins.

The descriptor is a n-dimensional vector which summarizes the gradient magnitude and orientation trend in a region around the keypoint location. In Lowe's implementation, the region is divided into 4 × 4 sub-regions, also called bins, and an orientation histogram is computed for each one. Each histogram has 8 orientation bins, which accumulate the gradient magnitude values referring to the canonical orientation. Therefore, the descriptor has a dimension of 128 (Figure 1).

The magnitude value of each pixel in the region is weighted by a Gaussian function and it is assigned to the corresponding sub-region. Moreover, the weighted magnitude value is accumulated in the orientation histogram. The purpose of the Gaussian window is to avoid sudden changes in the descriptor due to small changes in the position of the window, and to give less emphasis to gradients that are far from the centre of the descriptor. Finally, image contrast variations are removed by normalization to the unit length in order to make the descriptor invariant to contrast changes. Correspondence between two candidate points is found from an evaluation of the minimum distance between the n-dimensional vectors. The Euclidean distance is used. Lowe [11] proposed a matching technique in which the distance of the closest neighbour is compared to the distance of the second closest one. A stable matching is obtained if the rate between the second distance and the first one is more than the threshold value *Th_eu*. Lowe proposed *Th_eu =* 1.25, this means that the difference between the two distances must be more than 25% of the closest distance one.

## 3. Testing Methodology

The SIFT algorithm has been tested on a set of images and compared with the performances of the traditional feature extraction and matching algorithms used in photogrammetry. The Forstner operator [9], the Cross Correlation (CC), and the Least Square Matching technique [32] were used for the comparison analysis of the feature extraction and matching. These algorithms are implemented in commercial photogrammetric software and many researches have demonstrated their high reliability, especially as far as accuracy, which can reach sub-pixel dimensions, is concerned. Thus, these techniques can be considered suitable for comparison with the SIFT analysis in the photogrammetric field.

The SIFT algorithm is available on the Internet in many implementations [41-44]. These are demo versions, which can be used for repeatability and local accuracy tests on trial images; therefore, they cannot work with high resolution images. In order to overcome this limitation, the authors have developed their own implementation [45] of Lowe's original detector/descriptor algorithm in Matlab code. Then, in order to obtain a complete and reliable comparison, the Forstner operator, CC and LSM algorithms have also been implemented in Matlab.

Many criteria for the performance assessment of the feature extractors and matching techniques have been mentioned in literature. Many different variables such as 3D viewpoint changes or radiometric distortions can affect image acquisition; the definition of a suitable criterion for the estimation of the best technique is therefore not possible. Existing methods can be categorized as methods based on [30]: ground-truth verification, visual inspection, repeatability, localization accuracy, information content, theoretical analysis and specific tasks. Obviously, the performance of an interest operator varies in relation to the criterion that is adopted; an unambiguous evaluation of the most suitable feature extractor is therefore not available.

In this paper, the SIFT operator is compared to traditional techniques, considering the localization accuracy. This criterion evaluates the performance of a feature extraction and matching technique through the stereoscopic reconstruction accuracy of homologous points. The Least Median Square (LMS) [46] technique has been implemented (Matlab code) for robust relative orientation estimation, in order to detect the total number of homologous points extracted, the rate of mismatches (outliers) and the accuracy of the residual homologous points. In this way, it has been possible to evaluate the performances of both the extractors and the matching algorithms. The LMS algorithm removes outliers by means of a two step standard residual analysis according to the rejection threshold *L* [47]. Nevertheless, it is not an efficient estimator, and it does not supply accurate solutions. Therefore, the unknown parameters must be re-estimated using the Least Square estimator. The reliability of the relative orientation has been guaranteed by a homogenous distribution of points over the entire images [48]. Then, the auto adaptive version of the SIFT has been evaluated on different image pairs, comparing the achieved results with the original implementations.

The congruency of the relative orientation parameters has been checked in each test through a statistical comparison with a set of reference parameters. For this purpose, a relative orientation was carried out using manual stereoscopic measurements. The theoretical standard deviations of the angular parameters can be computed through the application of the variance-covariance propagation law to the Hallert equations [49]. The check analysis was carried out by comparing the difference of the mean of the angular parameters computed with automatic collimations, with the reference case ones. According to Krauss [49], we have evaluated if all the orientation values lie within the confidence interval (± 3*σ*).

## 4. Valitation Test

A validation test has been carried out in order to validate the algorithms and make first comparisons and considerations between the SIFT and LSM techniques. The test was performed on an aerial image pair acquired using an Intergraph Z/I Imaging DMC digital camera. The images were taken over a hilly wooded area near Lauria, a town in Basilicata (Italy). In order to speed up the automated computations, the original images (2,116 dpi), were resampled to a lower resolution, which corresponds to an equivalent flight altitude of 3,400 m, that is considered suitable for a Medium Scale map (1:10,000). The images were first pre-processed with the Wallis filter [50], in order to enhance the dynamic range of the image and to improve the local image quality, by enlarging the contrast around the edges. Then the Forstner extractor was applied, setting its threshold parameters according to Krauss's specifications. A Cross-Correlation technique was carried out with NCC = 0.8-0.9 and LSM was performed for the automatic collimation refinement.

The local detector thresholds were set in SIFT according to Lowe's original implementation and applied to images, while the rate of the Euclidean distance between the first and the second candidate to match was varied in the *Th*_*eu* = 1.25 – 2 range. Both the *Th_eu* and *NCC* parameters quantified the similarity between the matching correspondences. Finally, the LMS algorithm was tested on the image pair with increasing values of *L*. A suitable range, that was experimentally obtained, is *L=* 3-20. The experimental results are summarized in Table 1. A set of four rejection thresholds is shown in the main rows of the table, while the two main columns represent the results obtained using the two operators. *Mth* denotes the number of homologous pairs extracted and matched; *Mthok* and *%* represent the number and rate of the pairs which are not affected by blunders, respectively, while *p_max_* represents the maximum residual parallax in the pixel.

The experimental results allowed further considerations to be drawn: first, the similarity constraints of the SIFT and ForLSM techniques (*Th_eu, NCC*) affect the matching performances in different ways. On one hand, SIFT is not sensitive to the *Th_eu* threshold. On the other hand, the *NCC* value influences the performances of ForLSM at least in terms of number of matched points; the number of points extracted by *NCC* drastically decreases for higher values.

**Figure 2.**
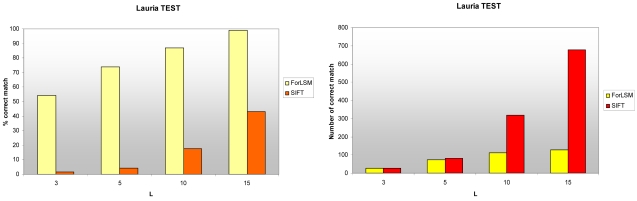
The rate and total number of correct correspondences found with ForLSM and SIFT in the Lauria test images, for different rejection threshold values.

In this test, the rate of matched point is always better in the ForLSM technique. The rate even reaches 98.9%, while SIFT cannot even reach 45% (Figure 2). Nevertheless, the number of corresponding points produced by SIFT and ForLSM is similar for low rejection thresholds, but the difference rises rapidly with the increase in the *L* values. Therefore, it is possible to state that SIFT matches more points than ForLSM (Figure 2). This aspect is of primary importance for the generation of an approximate DSM: the more points, the more accurate this model is and the better the approximation in the matching algorithms is.

The maximum residual parallaxes of the relative orientation obtained by SIFT and ForLSM are comparable and have a sub-pixel value in both cases. Although this result is quite different from previous works [31], it shows that region detectors can also achieve excellent performances in accuracy.

The performances of the two operators also depend on the criterion that is used for the blunder detection. In this case, the LMS estimation of the relative orientation using classical equations makes the selection sensitive to the rejection threshold of the LSM and to the interior parameters of the camera.

The congruency of the relative orientation parameters was established by comparing the difference in mean of the angular parameters, computed by automatic collimations of the different rejection thresholds, with the reference case ones. As can be seen in Table 2, all the orientation values are within the confidence interval (± 3*σ*). Therefore, these results allow a correct validation performance analysis of the two image matching methods to be carried out.

## 5. Practical Tests

Other tests on terrestrial and aerial images have been performed with the aim of evaluating the accuracy location. Terrestrial images were acquired using a non conventional taking geometry; aerial tests considered images acquired using the Politecnico di Torino mini-UAV. Some of the tests, which have been considered important, are presented.

### 5.1. Terrestrial images

**Figure 3.**
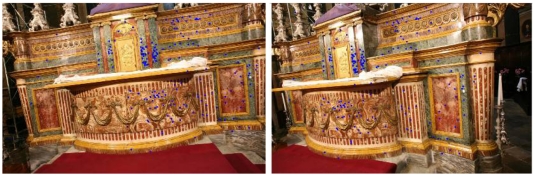
The Misericordia test. The image correspondences automatically matched with SIFT (303 points, *Th_eu* = 2, *L* = 10).

SIFT and ForLSM have been tested on close-range images with high geometrical distortions. The first stereoscopic pair was acquired in the Misericordia church in Turin (Italy) with a Canon EOS 5D camera, which had been calibrated with the *Calibra* software implemented by the Politecnico di Torino research group. The acquisition was performed in good illumination conditions; nevertheless, the images show a geometrical distortion due to a change in viewpoint of about 25° (Figure 3).

The evaluation of the matching methods was performed by fixing the *NCC* and T*h_eu* thresholds to 0.9 and 2, respetcively. The statistical analysis of the angular estimated parameters was then carried out. The experimental results are shown in Table 3.

It can be noticed that SIFT offers an excellent performance in terms of number of pairs extracted (643), a number which is almost five times higher than the number of pairs extracted using ForLSM (144). Furthermore, the rate of correctly matched points (*Mthok*) is quite stable in SIFT compared to the previous test, while it is rather reduced in ForLSM. The maximum parallaxes maintain sub-pixel values in SIFT for high rejection thresholds, while they are drastically increased in ForLSM. Furthermore, the statistical check of the ForLSM relative orientation has confirmed that the robust estimation is still affected by outliers; in fact the angular parameters do not satisfy the statistical equality with a reference set, for rejection threshold higher than 5.

**Figure 4.**
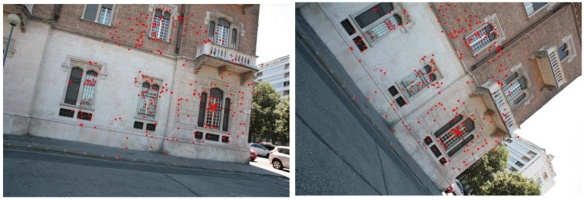
The Duca house test. The image correspondences automatically matched with SIFT (344 points, *Th_eu* = 2, *L* = 10).

Another test on terrestrial images has been performed in order to check the invariance to rotations of the SIFT technique. The acquisition was carried out on the façade of a house (Duca house) in Turin (Italy), with a Canon EOS 5D camera. Two images, with a rotation of about 40°, were taken and processed with ForLSM and SIFT (Figure 4).

Due to the high rotation, ForLSM did not produce stable matched pairs. The LMS estimator in fact had convergence problems for the whole range of the rejection threshold. SIFT, instead, gave very interesting results. Up to 600 stable pairs were extracted and matched, with residual parallaxes of less than 0.5 pixels (Table 4).

### 5.2. UAV images

**Figure 5.**
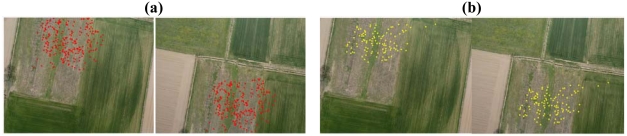
(a) The Villareggia test 1. The image correspondences automatically matched by SIFT (256 points, *Th_key* = 0.0005, *L* = 10). (b) The homologous points detected by ForLSM (100 points, *NCC* = 0.8, *L* = 10).

Performing fully automated stereoscopic image acquisition from a mini-UAV platform is currently a rather difficult task. Many different variables can affect flight stability, hence stereoscopic coverage is not always assured. Moreover, the image geometry seldom observes the normal condition. These conditions could cause some problems in the photogrammetric process, in particular in the automated image matching phase for tie point extractions, bundle block adjustment and approximate DSM generation. Therefore, an evaluation of the most suitable feature detectors and image matching techniques is undoubtedly necessary. A first evaluation test of the SIFT and ForLSM techniques was carried out on a image pair, acquired with the low-cost Pelican mini-UAV, which was developed by the research teams at the Land, Environment and Geo-engineering and Aerospace Engineering Departments of the Politecnico di Torino [51]. The image acquisition was carried out with a RICOH GR camera installed onboard, over an area in the countryside around the flight site in Villareggia (Turin, Italy), at a relative altitude of 100 m (Ground Sample Distance *=*0.04 m). The covered area consists of fields and meadows (Figure 5).

The same procedure that was used for the tests carried out on the close-range images was followed. The results provided by the SIFT and ForLSM methods are different from those of the previous tests (Table 5).

The number of pairs (Mth) extracted by SIFT (40) is lower than in the other tests. This difference is due to the setting of the threshold parameters of the detector. The thresholds recommended by Lowe give good results in terrestrial applications, where images are well-textured; but must be carefully set in aerial cases, such as this one, in which repetitive patterns or a lack of texture are possible. Many different trials were therefore performed on these images, in order to define the most suitable thresholds. The performance of the detector in relation to the *th_key* threshold was investigated. The experimental tests induced the authors to vary these parameters from Lowe's standard value (0.001) to 0.0005, which allowed 478 image pairs, with sub-pixel accuracy, to be detected.

The low stereoscopic coverage and the instability of the camera calibration sometimes caused problems for the LMS algorithm which had problems selecting the correct homologous points.

The RICOH camera, that was used, has a retractable lens system; therefore, an auto-calibration is needed before each image acquisition. In this case, the calibration carried out with the *Calibra* software was used, because it assured the best results in comparison with the auto-calibrations performed in the photogrammetric tests flights. Nevertheless, these problems are still apparent in the non-homogenous distribution of the points in the overlap area. The keypoints on the border of the stereoscopic images in particular were completely removed. This is a serious problem, as this ill-posed geometry compromises the convergence of the relative orientation, reduces the area of the approximate DSM and limits the information provided by the extracted tie-points in the bundle block adjustment.

A second test has been performed on a stereoscopic pair acquired over a ploughed field and a meadow, during the same flight as the previous test. In this case, the high repetitive patterns and a drag effect on one of the two images caused many problems in the image matching for both techniques. For this reason, the ForLSM did not managed to homologous points in the ploughed area, due to the bad texture.

**Figure 6.**
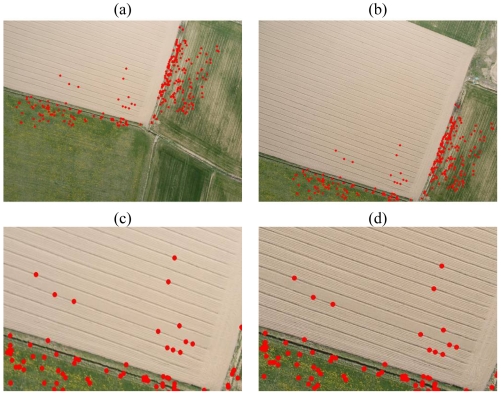
The Villareggia Test 2. (a), (b) The image correspondences automatically matched by SIFT (283 points, *D*|(*x̂*| = 0.01, *L* = 10). (c), (d) Details of the correspondences in the ploughed area.

SIFT, instead, furnished interesting results after a careful setting of the threshold parameters. In the first set of experiments, the *Th_key* threshold was varied over a wide range, but no interesting results were obtained. Other investigations on the contrast value D|(*x̂*| were therefore performed. As already mentioned, this parameter allows points to be removed in poor-contrast regions and it was set at 0.03, according to Lowe. In this application (Table 6), the reduction of D|(*x̂*| to 0.01 allowed a high number of points (457) to be extracted and matched with sub-pixel accuracy, in comparison to ForLSM (102). Furthermore, SIFT was able to detect some homologous points in the ploughed field (Figure 6), a situation where the collimation of correspondences is a very difficult task, even in manual mode. The high performances of SIFT on the images with a high rate of repetitive patterns, was due to the size of the descriptor, which is not restricted to the keypoint neighbourhood. In fact for high scales, the size can reach 100×100 pixels, therefore gradient information on well-textured zones near the keypoint can be detected.

A high number of extracted points determines in general a higher number of mismatches; this aspect, added to the reduced overlap and the camera calibration problems, can increase the instability of the LMS algorithm. On the other hand, this feature allows a more omogeonous distribution of the points and the generation of a better approximate DSM.

## 6. Auto-Adaptive SIFT Implementation (A^2^-SIFT)

The distribution of the features extracted on the image is a fundamental aspect for the relative orientation: points that are too close or blank areas can compromise the stability of the relative estimation between images. The number of points alone does not assure the quality of the image orientation. It has been shown that the SIFT operator can match a high number of points on well-textured areas, while it does not allow any points to be matched on poor textures. This problem affects UAV images, in particular, as they are usually acquired on non-urban areas, characterized by a homogeoneous land cover.

In the previous sections, a change in the threshold values allowed this problem to be partially solved: good results were achieved by modifying the contrast value. D|(*x̂*| It has been shown that decreasing its value, the number of extracted and matched points improves over the entire image and in particular over bad-textured areas. Nevertheless, this modification usually causes an improvement of the mismatch number and, as a consequence, decreases the stability of the relative orientation.

Furthermore, the setting of optimal thresholds is not always an easy task, and it requires several preliminary tests for each image in order to define the best values. In addition, a texture can vary on an image, as shown in Figure 6, where the texture is higher on the grass and it is therefore easier to match points there than in the ploughed area. In this situation, the keypoint extraction occurs irregularly and the use of a unique *D*|(*x̂*| does not solve this problem. In order to overcome this problem, a more complete study of local texture is necessary with the aim of extracting the keypoints over the entire image in a more uniform way.

For this reason, the SIFT algorithm was modified in order to fit the *D*|*x̂*| threshold according to the texture computed in the proximity of each keypoint: in other words, each keypoint has a different *D*|*x̂*| according to the texture of the image in its neighbourhood.

In order to do this, a coefficient (*Tx_coef*) which is able to define the local textue of the image was implemented. As known the SIFT operator extracts the keypoints from the DoG Scale Space; for this reason, the *Tx_coef* was obtained analysing this scale space. Similar implementations have already given good results in the texture analysis of grey level images [52]. This coefficient is equal to:

(3) $$Tx\_\text{coe}f=\frac{\overset{i=m+\text{rad}}{\underset{i=m-\text{rad}}{\text{∑}}}\overset{j=m-\text{rad}}{\underset{j=m-\text{rad}}{\text{∑}}}\sqrt{\frac{1}{N^{2}}\overset{k=i+\frac{\left(N-2\right)}{2}}{\underset{k=i-\frac{\left(N-1\right)}{2}}{\text{∑}}}\overset{l=j+\frac{\left(N-2\right)}{2}}{\underset{l=j-\frac{\left(N-2\right)}{2}}{\text{∑}}}\left(x_{kl}-{\bar{x}}_{N}\right)}}{4\cdot rad^{2}}$$

*m, n* are the coordinates of the keypoint in the image, *N* is equal to 7 pixels, *x_kl_* is the grey level value of the pixels in the *DoG* scale space and x̄_N_ is the mean grey level value in the *N*-by-*N* template. Finally, *rad* depends on the image scale and it is equal to:

(4) $$\text{rad}=4\cdot \sigma_{o}\cdot 2^{{(\text{s}}_{\text{i}}/\text{Scale})\cdot 2}$$

*s_i_* is the scale of the i-th keypoint, *Scale* is the total number of images in the *DoG* scale space and *σ_0_* is the prior smoothing [10]. The equation shows that the analysed area of each keypoint varies according to the scale space where it has been extracted.

This texture coefficient allows the local texture of the *DoG* scale around a keypoint to be evaluated: if the texture is good, the *Tx_coef* will be high and viceversa. In this way, it is possible to predict the image areas where the keypoint extraction is more difficult. As a conseguence, a lower contrast value *D*|(*x̂*)| can be used in these areas to extract a higher number of keypoints. And viceversa, a higher contrast value *D*|(*x̂*)| must be adopted in well-textured areas.

Several tests were performed on different areas characterized by a homogeneous texture; images acquired by the UAV were analysed in particular.

The *Tx_coef* and the number of extracted keypoints were considered for each of these areas, according to different *D*|(*x̂*)| values: in this way, it was possible to define a suitable contrast value *D*|(*x̂*)| for each texture. Several land cover typologies were considered (ploughed areas, grass areas built up areas, etc.), considering a set of UAV images. A suitable contrast value *D*|(*x̂*)| was evaluated for each typology.

In general, the *Tx_coef* value varies from 10^-1^ and 10^-4^ and this value is less than 10^-2^ in bad-textured areas. In this way, it was possible to define five different levels of the contrast value *D*|(*x̂*)| in a range between 0.01 and 0.05, according to their texture coefficient.

A set of different images was considered in order to validate this method; a comparison was made between the original SIFT implementation and the modified SIFT implementation. The tests were performed varying the contrast value *D*|(*x̂*)| in the original implementation and fixing all the other parameters (*Th_key, Th_eu*, etc.).

**Figure 7.**
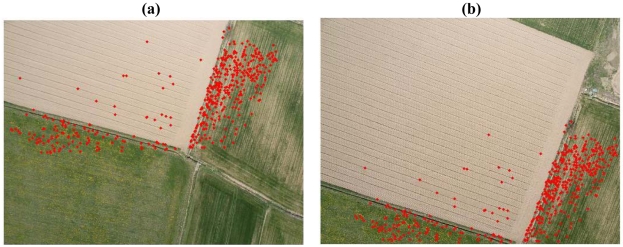
Villareggia Test 2. (a), (b) Image correspondences automatically matched by A^2^ SIFT (487 points, L = 10).

In the first test the image pair in Section 5.2 was considered (Figure 6). The achieved results have shown how the auto-adaptive version can detect a higher number of points all over the image and in particular in the ploughed area, where 21 points have been matched (Figure 7). The matched keypoints are extracted all over the image overlap.

The rate of mismatches is constant only for high *L* values, while A^2^ SIFT shows a higher rate in correspondence of lower *L* (Table 7). This problem is influenced by the camera calibration which conditions the rejection of keypoints on the boundary of the image. Nevertheless, the high mismatch rate does not influence the convergence of the relative orientation and the maxima parallaxes are lower in all the rejection levels.

Then, image pairs, characterized by different textures are considered, as the previous example. These images have bad-textures, but none repetitive patterns are visible. Also in this case, the difference given by the auto-adaptive in the distribution of points and in the relative orientation results is evaluated.

**Figure 8.**
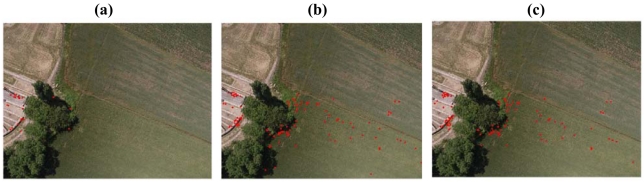
Comparative test. Image correspondences automatically matched (L=10) by SIFT with *D*|(*x̂*)|=0.01 (a), *D*|(*x̂*)|=0.03 (b), A^2^ SIFT (c).

The comparison of the auto-adaptive solution with the points extracted using *D*|(*x̂*)| = 0.03 and *D*|(*x̂*| = 0.01 is reported in Figure 8. Only one image of the stereopair is reported in order to evaluate the points' distribution.

The results shows that the adaptive solution gave a better distribution respect to the *D*|(*x̂*)| = 0.03 solution: in particular, the traditional implementation does not allow matching points all over the image and, for this reason, the relative orientation failed (see Table 8).

In contrast, the results achieved by *D*|(*x̂*)| =0.01 and by the auto-adaptive solution are similar: the number of correctly matched points and the distribution on the image are approximately the same. The auto-adaptive solution extracts and match less points than the *D*|(*x̂*)| = 0.01 solution; however, it shows a better percentage of correct matches.

In general, the A^2^ SIFT showed better results than *D*|(*x̂*)| = 0.03 implementation in all the performed tests. Furthermore, this difference is minimal if compared to the *D*|(*x̂*)| = 0.01 solution. In these cases, the differences were detectable only in images characterized by very bad textures, with repetitive patterns. Nevertheless, the distribution of extracted points is not ameliorated by the adaptive implementation and clusters of points are not avoided.

## 7. Conclusions and Future Developments

The tests presented in this paper highlight the good performance that can be obtained with the SIFT operator in feature extraction and matching in critical conditions. A comparison with algorithms commonly used in the photogrammetric community (Forstner operator, LSM technique) has underlined the capacity of SIFT to extract and match homologous points on image pairs with large geometric and photometric distortions. The huge number and the high accuracy of the correspondences confirm the SIFT invariance to wide rotations and projective distortions in a range limited to 20°-30°, as established by Lowe [11]. Then, the great advantage of this operator, compared to conventional techniques (LSM), is the possibility to reach good results without any approximate solutions. Although the computational time is quite higher than that of other techniques, SIFT can be considered a potential solution for the automatic tie point extraction in new photogrammetric applications, such as MMT systems, oblique cameras and UAV platforms.

Furthermore, the number of pairs extracted using SIFT is usually higher than the ones located by traditional techniques. This aspect is very important in the DSM generation: tie-points are useful in the image orientation and they provide an approximate DSM that is essential in image matching. The high number of points extracted by SIFT could speed-up these algorithms.

The features, however, are not always well distributed over the entire image, because of bad textured areas. This problem occurs above all in large scale aerial applications, where the standard threshold parameters suggested by Lowe for the detector algorithm do not provide reliable results. Experimental tests on images acquired by the mini-UAV shows that the thresholds of the local extrema value (*Th_key)* and the contrast value *D*|(*x̂*)| affects the final results in terms of precision and number of extracted points. In particular, the setting of the contrast threshold is strictly required, if the texture of the images is bad.

For this reason, a new auto adaptive implementation of the SIFT operator is realized in order to fit the *D*|(*x̂*)| threshold according to the texture computed in proximity of each keypoint. The auto adaptive SIFT is set for the UAV images that had shown to particularly suffers from this problem.

Achieved results have shown that the A^2^ SIFT gives always better results than *D*|(*x̂*)| =0.03 traditional implementation, in terms of extracted points and stability of the relative orientation. The comparison with *D*|(*x̂*)| =0.01 D implementation has instead shown little differences. However, the differences are stressed in presence of repetitive patterns; in these land cover typologies, the adaptive implementation can match the double of points.

In conclusion, the A^2^ SIFT improves the performances of the Lowe's implementation. The technique allows the automatic extraction of homologous points not only in presence of high geometric distortions, but also over bad textured images, where the traditional implementation usually fails. Therefore, A^2^ SIFT can be used in aerial photogrammetric applications over non-urban areas. The tie point extraction and the approximate DSM generation can be carried out in a complete automatic way without any visual inspection for the threshold setting.

## Figures and Tables

**Table 1.** The experimental results of the examined operators, in relation to the *NCC, Th_eu* and *L* parameters.

	**ForLSM**					**SIFT**				
		
**L**	**NCC**	**Mth**	**Mthok**	**%**	**p_max_ (pixel)**	**Th_eu**	**Mth**	**Mthok**	**%**	**p_max_ (pixel)**
**3**	0.9	46	25	54.3	0.088	1.25	2454	22	0.9	0.003
	0.85	92	25	27.2	0.046	1.5	2122	26	1.2	0.007
	0.8	134	27	20.1	0.036	2	1572	25	1.6	0.006
**5**	0.9	46	34	73.9	0.192	1.25	2454	76	3.1	0.013
	0.85	92	50	54.3	0.114	1.5	2122	81	3.8	0.014
	0.8	134	73	54.5	0.115	2	1572	66	4.2	0.014
**10**	0.9	46	40	87.0	0.500	1.25	2454	309	12.6	0.056
	0.85	92	80	87.0	0.522	1.5	2122	319	15.0	0.062
	0.8	134	111	82.8	0.354	2	1572	280	17.8	0.072
**15**	0.9	46	44	95.7	0.616	1.25	2454	616	25.1	0.122
	0.85	92	91	98.9	0.732	1.5	2122	651	30.7	0.148
	0.8	134	128	95.5	0.968	2	1572	677	43.1	0.182

**Table 2.** The relative orientation parameters performed through manual and automatique techniques and their statistical comparison.

**L**	**Mthok**	**p_max_ (pixel)**	**dk_1_ (gon)**	**dk_2_ (gon)**	**dΦ_1_ (gon)**	**dΦ_2_ (gon)**	**dω_2_ (gon)**
Relative Orientation Parameters (Manual)							
	10	0.41	1.7488 ± 0.0397	1.8533 ± 0.0397	0.0195 ± 0.0163	0.0173 ± 0.0163	0.0185 ± 0.0200
Relative Orientation Parameters (LSM)							
Mean (gon)			1.7731	1.8682	0.0161	0.0185	-0.0180
Standard dev. (gon)			0.0022	0.0084	0.00063	0.0006	0.0008
Diff. in mean(gon)			0.0243	0.0159	-0.0033	0.0012	0.0005
Relative Orientation Parameters (SIFT)							
Mean (gon)			1.7819	1.855	0.0185	0.0185	-0.019
Standard dev. (gon)			0.0039	0.004	0.00144	0.0004	0.00031
Diff. in mean(gon)			0.0331	0.002		-0.001	-0.0005

**Table 3.** The experimental results of the examined operators, in relation to the *NCC, Th_eu* and *L* parameters.

	**Forstner+LSM**					**SIFT**				
		
**L**	**NCC**	**mth**	**mthok**	**%**	**p_max_ (pixel)**	**Th_eu**	**Mth**	**Mthok**	**%**	**p_max_ (pixel)**
**5**	0.9	256	98	38.2	5.24	2	1500	95	6.4	0.032
**8**	0.9	256	124	48.4	14.2	2	1500	206	13.8	0.091
**10**	0.9	256	131	51.1	9.41	2	1500	303	20.2	0.143
**15**	0.9	256	144	56.2	45.7	2	1500	643	42.9	0.349

**Table 4.** The experimental results of the SIFT operator, in relation to the *Th_eu* and *L* parameters.

	**SIFT**				
	
**L**	**Th_eu**	**Mth**	**Mthok**	**%**	**p_max_ (pixel)**
**5**	2	1353	91	6.7	0.123
**8**	2	1353	204	15.1	0.158
**10**	2	1353	344	25.4	0.246
**15**	2	1353	599	44.3	0.445

**Table 5.** The Villareggia test 1. The experimental results of the examined operators, in relation to the *NCC, Th_key* and *L* parameters.

	**FORSTNER+LSM**					**SIFT**				
	
**L**	**NCC**	**Mth**	**Mthok**	**%**	**p_max_ (pixel)**	**Th_key**	**Mth**	**mthok**	**%**	**p_max_ (pixel)**
**3**	0.9	29	20	69.0	0.042	0.0005	478	21	4.3	0.015
	0.8	111	57	51.4	0.030	0.001	40	21	52.5	0.07
**5**	0.9	29	23	79.3	0.050	0.0005	478	61	12.7	0,013
	0.8	111	59	53.2	0.038	0.001	40	26	65.1	0.136
**10**	0.9	29	29	100.0	0.102	0.0005	478	256	53.5	0.06
	0.8	111	100	90.1	0.105	0.001	40	28	70.0	0.59
**15**	0.9	29	28	96.6	0.095	0.0005	478	366	76.5	0.131
	0.8	111	103	92.3	Noconv	0.001	40	29	72.5	0.636

**Table 6.** The Villareggia test 2. The experimental results of the examined operators, in relation to the *NCC, D*|(*x̂*)| and *L* parameters.

	**Forstner+LSM**					**SIFT**				
		
**L**	**NCC**	**Mth**	**Mthok**	**%**	**p_max_ (pixel)**	**|*D*(*x̂*)|**	**Mth**	**mthok**	**%**	**p_max_ (pixel)**
**3**	0.9	28	16	57.1	0.023	0.03	205	30	14.6	noconv
	0.8	182	36	19.7	0.148	0.01	745	35	4.7	noconv
**5**	0.9	28	20	71.4	0.067	0.03	205	76	37.0	noconv
	0.8	182	79	43.4	noconv	0.01	745	87	11.7	0.019
**10**	0.9	28	21	75.0	0.087	0.03	205	151	73.7	0.173
	0.8	182	99	54.4	noconv	0.01	745	283	38.0	0.082
**15**	0.9	28	23	82.1	0.172	0.03	205	177	86.3	0.112
	0.8	182	102	56.0	0.463	0.01	745	457	61.3	0.193

**Table 7.** Villareggia Test 2. Experimental results of the SIFT operator, in relation to, |*D*(*x̂*)**|** *L* parameters.

	**|*D*(*x̂*)| =0.01**				**A^2^ SIFT**			
		
**L**	**Mth**	**mthok**	**%**	**p_max_ (pixel)**	**Mth**	**mthok**	**%**	**p_max_ (pixel)**
**3**	745	35	4.7	noconv	1628	56	3.5	0.004
**5**	745	87	11.7	0.019	1628	124	7.6	0.021
**10**	745	283	38.0	0.082	1628	488	29.9	0.047
**15**	745	457	61.3	0.193	1628	990	60.9	0.137

**Table 8.** Comparative test. Experimental results of the SIFT operator, in relation to *D*|(*x̂*)| and *L* parameters.

	***D*|(*x̂*)| = 0.03**				***D*|(*x̂*)| = 0.01**				**A^2^ SIFT**			
			
**L**	**Mth**	**Mthok**	**%**	**p_max_ pixel)**	**Mth**	**mthok**	**%**	**p_max_(pixel)**	**Mth**	**mthok**	**%**	**p_max_(pixel)**
**3**	28	20	71.4	noconv	166	26	15.6	0.060	148	29	19.6	0.067
**5**	28	19	67.4	noconv	166	67	40.3	0.103	148	62	41.9	0.092
**10**	28	24	85.7	noconv	166	122	73.5	0.148	148	112	75.7	0.127
**15**	28	25	89.3	noconv	166	140	84.3	0.224	148	132	89.2	0.235

## References

[1] Se S., Lowe D.G., Little J. Vision-based mobile robot localization and mapping using scale-invariant features.

[2] Gordon I., Lowe D.G. (2006). What and where: 3D object recognition with accurate pose. Toward Category-Level Object Recognition.

[3] Brown M., Lowe D.G. Recognising panoramas.

[4] Rodehorst V., Koschan A. Comparison and evaluation of feature point detectors.

[5] Mikolajczyk K., Tuytelaars T., Schmid C., Zisserman A., Matas J., Schaffalitzky F., Kadir T., Van Gool L. (2005). A comparison of affine region detectors. Int. J. Comput. Vis..

[6] Bendea H., Cina A., De Agostino M., Lingua A., Piras M. Realizzazione di un GIS stradale con veicolo rilevatore basso costo.

[7] Poli D. (2006). Reality based 3D city models for aerial and satellite data. GEOInformatics.

[8] Bendea H., Chiabrando F., Giulio Tonolo F., Marenchino D. (2007). Mapping of archaeological areas using a low-cost uav. The augusta bagiennorum test site.

[9] Forstner W. (1986). A feature based correspondence algorithm for image matching.

[10] Harris C., Stephens M. (1988). A combined corner and edge detector.

[11] Lowe D. (2004). Distinctive image features from scale-invariant keypoints. Int. J. Comput. Vis..

[12] Sivic J., Zisserman A. (2003). Video Google: A text retrieval approach to object matching in videos.

[13] Delponte E., Isgrò F., Odone F., Verri A. (2006). SVD-matching using SIFT features. Graph. Models.

[14] Yun S.U., Min D., Sohn K. 3D scene reconstruction system with hand-held stereo cameras.

[15] Ogawa Y., Shimada N., Shirai Y. (2007). Environmental mapping for mobile robot by tracking SIFT feature points using trinocular vision.

[16] Ali S., Scovanner P., Shah M. (2007). A 3-Dimensional SIFT descriptor and its application to action recognition.

[17] Ostiak P. Implementation of HDR panorama stitching algorithm.

[18] Battiato S., Gallo G., Puglisi G., Scellato S. SIFT features tracking for Video Stabilization.

[19] Battiato S., Gallo G., Puglisi G., Scellato S. Improved feature-points tracking for video stabilization.

[20] Kalantari M., Kassera M. Implementation of a low-cost photogrammetric methodology for 3D modelling of ceramic fragments.

[21] Heinrichs M., Hellwich O., Rodehorst V. Robust spatio-temporal feature tracking.

[22] Fraundorfer F., Frahm J.M., Snoeyink J., Pollefeys M., Wu C. Image localization in satellite imagery with feature-based indexing.

[23] Yang Y., Newsam S. Comparing SIFT descriptors and Gabor texture features for classification of remote sensed imagery.

[24] Abedinia A., Hahnb M., Samadzadegana F. An investigation into the registration of LIDAR intensity data and aerial images using the SIFT approach.

[25] Li F., Zhang G., Yan J. Coregistration based on SIFT algorithm for synthetic aperture radar interferometry.

[26] Forstner W., Steffen R. On visual real time mapping for Unmanned Aerial Vehicles.

[27] Ke Y., Sukthankar R. (2004). PCA-SIFT: A more distinctive representation for local image descriptors.

[28] Bay H., Ess A., Tuytelaars T., Van Gool L. (2004). Speeded-up robust features (SURF). J. Comput. Vis. Image Underst..

[29] Mikolajczyk K., Schmid C. (2005). A performance evaluation of local descriptors. IEEE Trans. Pattern Anal. Mach. Intell..

[30] Schmid C., Mohr R., Bauckhage C. (2000). Evaluation of interest point detectors. Int. J. Comput. Vis..

[31] Remondino F. (2006). Detectors and descriptors for photogrammetric applications.

[32] Ackermann F. (1984). Digital image correlation: Performance and potential application in photogrammetry. Photogram. Rec..

[33] Zhang L. (2005). Automatic Digital Surface Model (DSM) generation from linear array images. Thesis Dissertation, ETH No. 16078.

[34] Baltsavias E. (1991). Multiphoto Geometrically Constrained Matching. PhD. Dissertation..

[35] Lowe D.G. (2004). Object recognition from local scale-invariant features.

[36] Koenderink J.J. (1984). The structure of images. Biol. Cybern..

[37] Lindeberg T. (1988). Feature detection with automatic scale selection. Int. J. Comput. Vis..

[38] Brown M., Lowe D.G. (2002). Invariant features from interest points groups.

[39] Johnson A., Hebert M. (1997). Object recognition by matching oriented points.

[40] Zabih R., Woodfill J. (1994). Non-parametric local transforms for computing visual correspondence.

[41] Nowozin S. Libsift - Scale-invariant feature transform implementation. http://user.cs.tu-berlin.de/~nowozin/libsift.

[42] SIFT for Matlab http://vision.ucla.edu/~vedaldi/code/sift/sift.html.

[43] Demo Software: SIFT Keypoint Detector http://www.cs.ubc.ca/~lowe/keypoints/.

[44] Affine Covariant Features http://www.robots.ox.ac.uk/~vgg/research/affine/.

[45] Lingua A., Marenchino D., Nex F. L'operatore SIFT per l'orientamento di immagini acquisite con prese non-normali.

[46] Rousseeuw P.J., Leroy A.M. (1987). Robust regression and outlier detection.

[47] Bellone T., Lingua A., Rinaudo F. (1999). Tecniche di stima robusta in fotogrammetria digitale.

[48] Von Gruber O. (1924). Einfache und Doppelpunkteinschaltung im Raum.

[49] Kraus K. (1993). Photogrammetry, Theory and applications.

[50] Wallis R. An approach to the space variant restoration and enhancement of images.

[51] Boccardo P., Dequal S., Giulio Tonolo F., Marenchino D. (2006). ITHACA: Un progetto innovativo per la gestione delle emergenze ambientali.

[52] Nex F., Rinaudo F. Multi-image matching: An “Old And New” photogrammetric answer to lidar techniques.

